# The impact of participant mental health on attendance and engagement in a trial of behavioural weight management programmes: secondary analysis of the WRAP randomised controlled trial

**DOI:** 10.1186/s12966-021-01216-6

**Published:** 2021-11-07

**Authors:** Rebecca A. Jones, Julia Mueller, Stephen J. Sharp, Ann Vincent, Robbie Duschinsky, Simon J. Griffin, Amy L. Ahern

**Affiliations:** 1 MRC Epidemiology Unit, University of Cambridge, Cambridge, UK; 2 Department of Medicine, University College London, London, UK; 3 Primary Care Unit, Department of Public Health and Primary Care, University of Cambridge, Cambridge, UK

**Keywords:** Obesity, Prevention, Weight loss, Mental health, Engagement

## Abstract

**Background:**

Low attendance and engagement in behavioural weight management trials are common. Mental health may play an important role, however previous research exploring this association is limited with inconsistent findings. We aimed to investigate whether mental health was associated with attendance and engagement in a trial of behavioural weight management programmes.

**Methods:**

This is a secondary data analysis of the Weight loss referrals for adults in primary care (WRAP) trial, which randomised 1267 adults with overweight or obesity to brief intervention, WW (formerly Weight Watchers) for 12-weeks, or WW for 52-weeks. We used regression analyses to assess the association of baseline mental health (depression and anxiety (by Hospital Anxiety and Depression Scale), quality of life (by EQ5D), satisfaction with life (by Satisfaction with Life Questionnaire)) with programme attendance and engagement in WW groups, and trial attendance in all randomised groups.

**Results:**

Every one unit of baseline depression score was associated with a 1% relative reduction in rate of WW session attendance in the first 12 weeks (Incidence rate ratio [IRR] 0.99; 95% CI 0.98, 0.999). Higher baseline anxiety was associated with 4% lower odds to report high engagement with WW digital tools (Odds ratio [OR] 0.96; 95% CI 0.94, 0.99). Every one unit of global quality of life was associated with 69% lower odds of reporting high engagement with the WW mobile app (OR 0.31; 95% CI 0.15, 0.64). Greater symptoms of depression and anxiety and lower satisfaction with life at baseline were consistently associated with lower odds of attending study visits at 3-, 12-, 24-, and 60-months.

**Conclusions:**

Participants were less likely to attend programme sessions, engage with resources, and attend study assessments when reporting poorer baseline mental health. Differences in attendance and engagement were small, however changes may still have a meaningful effect on programme effectiveness and trial completion. Future research should investigate strategies to maximise attendance and engagement in those reporting poorer mental health.

**Trial registration:**

The original trial (ISRCTN82857232) and five year follow up (ISRCTN64986150) were prospectively registered with Current Controlled Trials on 15/10/2012 and 01/02/2018.

**Supplementary Information:**

The online version contains supplementary material available at 10.1186/s12966-021-01216-6.

## Background

Behavioural weight management programmes are internationally recommended practice for the treatment of overweight and obesity [1]. Good evidence shows that behavioural programmes benefit physical health [2–5], and a recent systematic review suggests there may be small benefits for some aspects of mental health, with no evidence to suggest that programmes negatively impact mental health [6]. However, low attendance and engagement with these programmes are common [7, 8], and the reasons for this are poorly understood. Low attendance and engagement can decrease the opportunity for participants to gain the skills, strategies, knowledge, and social support offered by the weight management programmes [8]. Previous research has reported that low levels of attendance and engagement in behavioural weight management programmes are associated with decreased likelihood of achieving clinically significant weight loss, consequently reducing the likelihood of gaining the associated health benefits [9–11], highlighting the importance of better understanding the reasons for poor attendance and engagement.

Previous research has sought to identify factors associated with attendance and engagement in behavioural weight management trials, however, findings have been constrained by the limited diversity of potentially associated factors assessed. Demographic factors, such as age, deprivation, and gender, have been commonly assessed for their association, with evidence suggesting better attendance and engagement among people who are older in age, more educated, and female [12–14]. However, previous research lacks sufficient investigation into how mental health may be associated with attendance and engagement.

Some researchers have suggested that mental health may play an important role in attendance and engagement of behavioural weight management programmes and in attrition of trials evaluating these programmes. There is a well-evidenced bidirectional relationship between obesity and mental health, with poor mental health being both a cause and consequence of obesity [15–20]. Additionally, both obesity and poor mental health are associated with experiencing stigma and discrimination, which is in turn associated with the avoidance of health promoting activities (e.g., behavioural weight management programmes) [21–23]. Furthermore, poor mental health can exacerbate feelings of amotivation [24–26] and can increase the likelihood to socially withdraw and isolate [27–29]. Thus, it is plausible that mental health may be associated with attendance and engagement with a behavioural weight management programme. Mental health is increasingly being considered as a symptom continuum which recognises that individuals can experience one or more symptoms of mental illness without meeting diagnostic criteria [30, 31]. Embracing the symptom continuum-based definition of mental health is associated with reduced stigma and improved attitudes toward mental ill-health [31, 32].

As stated, previous research lacks sufficient investigation into the role of mental health in attendance at and engagement with behavioural weight management programmes. The findings of the limited existing research findings are conflicting with some evidence reporting lower attendance and completion rates among weight management participants with higher levels of anxiety or depression [13, 33, 34], whilst other research reports mental health to not be associated with attendance and engagement [35]. A systematic review reported not finding any consistently associated psychological factors, though these findings were limited by the small number of studies assessing each factor [7]. The limited previous research and conflicting evidence highlights a need for further research investigating the relationship between mental health and attendance at and engagement with behavioural weight management programmes.

It is also plausible that mental health may influence whether a participant attends study follow-up visits. This is important as, although on average mental health appears to improve following a behavioural weight management programme [6], if baseline mental health is associated with attrition then it is possible that this finding may be biased by unrepresentative participant samples. Participant samples may be biased to include the most mentally healthy participants, and this would minimise the generalisability of evidence produced from weight management trials, particularly when assessing the impact of weight management programmes on mental health. We must investigate how participant mental health influences attendance at follow-up assessments for behavioural weight management trials to determine whether participant samples accurately reflect the true range of mental health experiences.

The current study aimed to investigate whether baseline mental health was associated with attendance and engagement with a behavioural weight management programme and completion of follow-up assessments in a randomised controlled trial. By better understanding the influence of baseline mental health on attendance and engagement, appropriate strategies may be implemented to support participation, minimise attrition, and subsequentially benefit health. In this study, we embrace the continuum-based definition of mental health which appreciates that individuals can experience one or more symptoms of mental illness without meeting diagnostic criteria [30, 31]. 

## Methods

### Study design

This study is a secondary data analysis of the Weight loss Referrals for Adults in Primary care (WRAP) trial, a non-blinded, multi-arm, randomised controlled trial comparing three intervention arms: (1) Brief intervention (BI), (2) 12-weeks commercial weight management programme (CP12), (3) 52-weeks commercial weight management programme (CP52). Participants who met eligibility criteria and gave informed consent were randomly assigned to an intervention arm on a 2:5:5 ratio with a block size of 12, stratified by research centre and gender. More detailed trial methods are reported elsewhere [36]. Ethical approval up to two year post randomisation follow up was received by East of England Cambridge East with local approvals from NRES Committee North West Liverpool Central and NRES Committee South Central Oxford. Ethical approval for five years post randomisation follow-up was received from West Midlands- Coventry and Warwickshire Research Ethics Committee 8th December 2017. The original trial (ISRCTN82857232) and 5 year follow up (ISRCTN64986150) were prospectively registered with Current Controlled Trials on 15/10/2012 and 01/02/2018 (10.1186/ISRCTN82857232;10.1186/ISRCTN64986150).

### Participants

Adults with a body mass index of 28 kg/m^2^ or greater were identified and recruited by local primary care practices across England. Exclusion criteria were: planned or current pregnancy in the next two years, previous or planned bariatric surgery, currently following a weight loss programme, non-English Speaking or communication needs that would preclude them from understanding the study materials and interventions, and general practitioner-defined inappropriate for participation (e.g., patients who are violent/terminally ill/have a history of an eating disorder). All participants gave written informed consent.

### Intervention

Participants randomly assigned to CP12 or CP52 were provided with vouchers to attend a weekly local WW (formerly Weight Watchers) meeting for the duration of the intervention they were assigned to (12-weeks or 52-weeks). Participants were provided with a unique code to access the WW digital tools for the duration of their assigned intervention. Participants assigned to the brief intervention control group were given a 32-page printed booklet by the British Heart Foundation of self-help weight-management strategies [37]. Research staff read a scripted booklet introduction to the participant.

### Outcomes

Study participants completed outcome assessments at baseline, 3-, 12-, 24- and 60-months. The outcomes of interest in this study were:

- Attendance at WW sessions (i.e., programme attendance): defined as the number of weekly WW sessions attended in the first 3 months of the programme (possible range of 0 to 12). Attendance at WW sessions were categorised as low (≤ 4 sessions), moderate (> 4 and ≤ 8 sessions), or high (> 8 and ≤ 12 sessions) attendance. Programme attendance was calculated by data collected by WW at weekly meetings (participants were provided booklets of vouchers to attend meetings, and WW reported how many vouchers were used). Data related to vouchers issued during a specific time period were not recorded due to a computer system error; as the only difference between those with and without data was referral date, missing data was considered to be missing at random.
- Engagement with WW digital tools (i.e., programme engagement): defined as (1) weekly frequency of use of the WW e-tools and online resources and (2) weekly frequency of use of the WW mobile phone app. The WW e-tools are an online service that includes access to support materials (e.g., recipes, videos, community area) and tracking tools. Engagement with digital tools was self-reported at 3-months from baseline, and answers were multiple choice (coded [1] Daily/almost daily, [2] 3–5 times per weeks, [3] 1–2 times per week, [4] Never/almost never).
- Study attendance (i.e., study visit attendance): defined as attendance at study clinic visits at 3-, 12-, 24- and 60-month follow-up assessments. Study attendance was monitored by the research team and categorised as (1) did attend visit or (2) did not attend visit.

Exposure variables were mental health-related measures at baseline, including quality of life (measured by EQ5D) [38], satisfaction with life (measured by the Satisfaction with Life Questionnaire (SLQ)) [39], and depression and anxiety (measured by the Hospital Anxiety and Depression Scale (HADS)) [40].

The EQ5D, used to measure quality of life, has 5 dimensions ([1] Mobility, [2] Self-care, [3] Usual activities, [4] Pain/discomfort, [5] Anxiety/depression) which are rated on 3 levels of severity (Level 1: No problem, Level 2: Some problems, Level 3: Extreme problems) [38]. Scores for the individual dimensions are collated to create a single index score; we used the UK value set and algorithm to calculate the index scores used. Potential index scores range from −0.281 to 1, where an index score less than 0 represents a state worse than death [41]. The EQ5D is a continuous measure derived from a validated questionnaire and is commonly used measure [38].

The Satisfaction with Life Questionnaire (SLQ) is a 5-item, 7-point scale ([1] strongly disagree to [7] strongly agree) that measures an individuals’ perception of their satisfaction with their life as a whole, allowing the individual to combine and weight aspects contributing to life satisfaction (e.g., health, finances, socialisation) according to their own criteria and judgement [39, 42, 43]. The SLQ score is calculated by adding the scores for each of the 5-items, with a higher total score representing a greater sense of life satisfaction [39]. The SLQ is a valid and reliable scale, with suitability for a wide range of population groups [42, 43].

The HADS measure is a validated screening tool for anxiety and depression with 14 items (1:1 ratio for anxiety:depression) scored on a Likert scale from 0 to 3 [40, 44, 45]. The scale produces symptom scores for anxiety and depression that range from 0 to 21, with a score of equal to or greater than 11 representing moderate to severe symptoms of depression and/or anxiety [40, 44, 45]. The scale cannot provide a clinical diagnosis of anxiety or depression [40, 44, 45]. The recent core outcome set for trials of behavioural adult weight management recommends the inclusion of the HADS measure to strengthen the consistency of reporting across trials [46].

### Statistical analysis

Stata (version 16; College Station, TX) [47] was used for all statistical analyses. The aims of this secondary analysis were determined a-priori and comprehensive statistical analyses plan determined prior to obtaining the trial data. Descriptive summary statistics were calculated for baseline characteristics by randomised group, with number of participants, mean, and standard deviation presented for continuous variables and number and proportion of participants presented for categorical variables.

#### Association of mental health with programme attendance and engagement

Negative binomial regression was used to estimate the effect of participant mental health on programme attendance, controlling for randomised group. Ordered logistic regression was used to estimate the effect on engagement with digital tools, controlling for randomised group. In the ordered logistic regression models, engagement response options were categorised as (1) Daily/Almost daily, (2) 3–5 times per week, (3) 1–2 times per week, and (4) Never/Almost never. Therefore, results were interpreted as the reduction in engagement associated with every unit change in mental health outcome. Analyses were performed using data from the intervention groups only (CP12 and CP52), and robust standard errors were calculated in all models to allow for clustering by general practice.

#### Association of mental health with study visit attendance

Logistic regression was used to estimate the association between baseline participant mental health and attendance at study follow-up visits (at 3-, 12-, 24-, and 60-months), controlling for randomised group and with robust standard errors to allow for clustering by general practice. Baseline mental health variables whose *p*-value for association with study visit attendance was < 0.05 were included in mutually adjusted models. Analyses were conducted using data from both intervention and control groups.

## Results

### Baseline characteristics

**Fig. 1:**
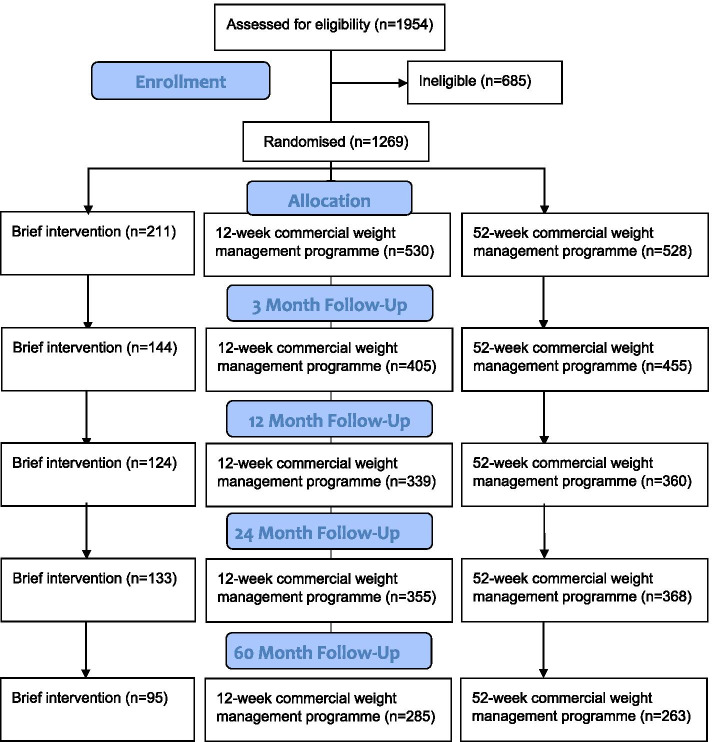
Participant flow diagram (CONSORT)

Between 18 October 2012 and 10 February 2014, 1269 eligible participants were randomised to one of three groups. Two participants were excluded after the baseline appointment and prior to the intervention started due to illnesses that rendered them ineligible for the trial. Figure 1 shows the participant flow through the trial. Participant characteristics at baseline are presented by randomised group in Table 1.

**Table 1:** Participant characteristics at baseline by randomised group

Baseline characteristics				
		BI (***n*** = 211)	CP12 (***n*** = 528)	CP52 (***n*** = 528)
**Age (years) [mean ± SD]**		51.91 ± 14.07	53.63 ± 13.26	53.30 ± 13.96
**Female**		143 (68%)	357 (68%)	359 (68%)
**Body mass index (BMI: kg/m**^**2**^**) [mean ± SD]**		34.43 ± 4.63	34.68 ± 5.39	34.45 ± 5.05
**Self-reports taking antidepressant medication**		26 (19%)	81 (21%)	81 (23%)
**Anxiety [mean ± SD]**		7.25 ± 4.29	6.89 ± 3.97	7.29 ± 4.09
**Depression [mean ± SD]**		5.58 ± 3.77	5.24 ± 3.38	5.20 ± 3.64
**Ethnicity**	**White or White British**	181 (86%)	480 (91%)	475 (90%)
	**Asian or Asian British**	9 (4%)	11 (2%)	15 (3%)
	**Black or Black British**	5 (2%)	12 (2%)	6 (1%)
	**Mixed or multiple ethnic group**	4 (2%)	4 (1%)	7 (1%)
	**Other, missing, or prefer not to say**	12 (6%)	21 (4%)	25 (4%)
**Index of Multiple Deprivation (IMD) decile**	**1 (least deprived)**	16 (8%)	45 (9%)	44 (8%)
	**2**	8 (4%)	16 (3%)	26 (5%)
	**3**	12 (6%)	38 (7%)	32 (6%)
	**4**	17 (8%)	37 (7%)	38 (8%)
	**5**	31 (15%)	49 (9%)	50 (10%)
	**6**	19 (9%)	61 (12%)	57 (11%)
	**7**	32 (15%)	67 (13%)	63 (12%)
	**8**	19 (9%)	69 (13%)	75 (14%)
	**9**	12 (6%)	51 (10%)	47 (9%)
	**10 (most deprived)**	45 (21%)	95 (18%)	94 (18%)
**Level of education**	**None**	7 (3.57%)	25 (5%)	27 (6%)
	**GCSE or equivalent**	55 (28%)	153 (32%)	155 (33%)
	**A-Level or equivalent**	53 (27%)	95 (20%)	110 (24%)
	**Post-secondary study**	10 (5%)	14 (3%)	10 (2%)
	**University degree or equivalent**	48 (24%)	108 (23%)	97 (21%)
	**Higher degree or equivalent**	23 (12%)	79 (17%)	68 (15%)
**Household income**	**£0–£19,999**	65 (33%)	125 (25%)	138 (28%)
	**£20,000–£49,999**	66 (33%)	173 (34%)	176 (35%)
	**£50,000+**	41 (21%)	91 (18%)	84 (17%)
	**Do not know / Prefer not to say**	27 (14%)	113 (23%)	101 (20%)

*Abbreviations: WW* Formerly Weight Watchers, *BI* Brief intervention, CP12–12 week commercial weight management programme (WW), CP52–52 week commercial weight management programme (WW), *SD* Standard deviation, *BMI* Body mass index, *GCSE* General Certificate of Secondary Education, A-Level – Advanced Level Qualification

On average, participants attended approximately 10 out of 12 WW sessions during the first 3-months of the study (12-week programme: 9.63 ± 3.44 sessions (80%); 52-week programme: 9.73 ± 3.41 sessions (81%)) (Table 2). Fifty-six percent of the 12-week programme participants and 50% of the 52-week programme participants never or almost never used the WW e-tools or online resources (Table 2). A high proportion of the study participants never or almost never used the WW mobile phone app (12-week programme: 80%; 52-week programme: 73%) (Table 2).

**Table 2:** Attendance and engagement with WW interventions, presented by intervention arm

WW session attendance and engagement with programme resources			
		CP12	CP52
**Attendance at WW sessions in first 3-months** *(CP12 (n = 272); CP52 (n = 360))*	**Mean number of sessions attended (± SD)**	9.63 ± 3.44	9.73 ± 3.41
	**Low attendance (n %) (≤4 sessions)**	35 (13%)	55 (15%)
	**Moderate attendance (n %) (> 4 & ≤ 8 sessions)**	33 (12%)	29 (8%)
	**High attendance (n %) (> 8 & ≤ 12 sessions)**	204 (75%)	276 (77%)
**Frequency of using WW e-tools/online resources** *(CP12 (n = 355); CP52 (n = 400))*	**Daily/Almost daily**	62 (17%)	91 (23%)
	**3–5 times per week**	30 (8%)	40 (10%)
	**1–2 times per week**	63 (18%)	67 (17%)
	**Never/Almost never**	200 (56%)	202 (51%)
**Frequency of using the WW mobile phone app** *(CP12 (n = 356); CP52 (n = 401))*	**Daily/Almost daily**	35 (10%)	60 (15%)
	**3–5 times per week**	15 (4%)	15 (4%)
	**1–2 times per week**	22 (6%)	32 (8%)
	**Never/Almost never**	284 (80%)	294 (73%)

*Abbreviations*: *WW* formerly Weight Watchers, *SD* Standard Deviation, CP12–12 week commercial weight management programme (WW), CP52–52 week commercial weight management programme (WW)

The number of participants completing follow-up assessments was 1004 participants at 3-months (79%), 823 participants at 12-months (65%), 856 participants at 24-months (68%), and 643 at 60-months (51%). Reasons for withdrawal from the study are detailed in Table 3.

**Table 3:** Reasons for withdrawal from the study, presented by randomised group

Withdrawal reasons			
	BI (***n*** = 58)	CP12 (***n*** = 110)	CP52 (***n*** = 100)
**Changed mind about participating**	12 (21%)	21 (19%)	28 (28%)
**Found another weight loss method**	1 (2%)	2 (2%)	1 (1%)
**Health issues**	5 (9%)	16 (15%)	15 (15%)
**Moved away**	7 (12%)	17 (15%)	13 (13%)
**No reason given**	6 (10%)	16 (15%)	9 (9%)
**Personal reasons**	7 (12%)	12 (11%)	14 (14%)
**Time/other commitments**	6 (10%)	13 (12%)	9 (9%)
**Trouble attending study visits**	0	3 (3%)	1 (1%)
**Unable to attend WW**	N/A	1 (1%)	2 (2%)
**Unhappy with study procedures**	0	2 (2%)	2 (2%)
**Unhappy with intervention**	14 (24%)	7 (6%)	6 (6%)

*Abbreviations: WW* Formerly Weight Watchers, *BI* Brief intervention, CP12–12 week commercial weight management programme (WW), CP52–52 week commercial weight management programme (WW)

### Association of mental health with programme attendance and engagement

#### Association of mental health with attendance at WW sessions

Baseline scores for global quality of life (Incidence rate ratio (IRR) 1.06; 95% confidence interval (CI) 0.96, 1.17; *n* = 608), anxiety (IRR 1.00; 95% CI 0.99, 1.01; *n* = 625) and satisfaction with life (IRR 1.00; 95% CI 0.999, 1.01; *n* = 620) were not associated with attendance at WW sessions in the first 3-months (Table 4). Every one unit of baseline depression score was associated with a 1% relative reduction in the rate of session attendance (IRR 0.99; 95% CI 0.98, 0.999; *n* = 625) (Table 4).

**Table 4:** Association of baseline mental health with attendance at WW sessions in first 3-months: Results are presented from negative binomial regression models, controlled for randomised group and with robust standard errors to allow for clustering by general practice

Attendance at WW sessions in first 3-months		
Mental health at baseline:	Incidence rate ratio (95% CI)	Number of participants
**Model 1: Global quality of life**	1.06 (0.96, 1.17)	608
**Model 2: Satisfaction with life**	1.00 (0.999, 1.01)	620
**Model 3: Anxiety**	1.00 (0.99, 1.01)	625
**Model 4: Depression**	0.99 (0.98, 0.999)	625

*Abbreviations: WW* Formerly Weight Watchers, *CI* Confidence interval

#### Association of mental health with programme engagement

Baseline scores for global quality of life (Odds Ratio (OR) 0.65; 95% CI 0.39, 1.09; *n* = 728), satisfaction with life (OR 1.01; 95% CI 0.99, 1.03; *n* = 743), and depression (OR 1.00; 95% CI 0.97, 1.04; *n* = 747) were not associated with weekly frequency of using WW e-tools/online resource (Table 5). Participants’ baseline score for anxiety was associated with self-reported engagement with the WW e-tools and online resources; every one unit of baseline anxiety was associated with 4% lower odds of reporting high levels of engagement with the WW e-tools and online resources (OR 0.96; 95% CI 0.94, 0.99; *n* = 747) (Table 5).

**Table 5:** Association of baseline mental health with weekly use of WW digital resources in first 3 months: Results are presented from negative binomial regression models, controlled for randomised group and with robust standard errors to allow for clustering by general practice

**Weekly frequency of using WW e-tools/online resources (reported at 3-months)**		
**Mental health at baseline:**	**Odds Ratio (95% CI)**	**Number of participants**
**Model 1: Global quality of life**	0.65 (0.39, 1.09)	728
**Model 2: Satisfaction with life**	1.01 (0.99, 1.03)	743
**Model 3: Anxiety**	0.96 (0.94, 0.99)	747
**Model 4: Depression**	1.00 (0.97, 1.04)	747
**Weekly frequency of using the WW mobile phone app (reported at 3-months)**		
**Mental health at baseline:**	**Odds Ratio (95% CI)**	**Number of participants**
**Model 1: Global quality of life**	0.31 (0.15, 0.64)	730
**Model 2: Satisfaction with life**	1.02 (0.996, 1.04)	745
**Model 3: Anxiety**	0.98 (0.95, 1.02)	749
**Model 4: Depression**	1.00 (0.96, 1.05)	749

*Abbreviations: WW* formerly Weight Watchers, *CI* Confidence interval

#### Association of mental health with engagement with WW mobile phone app

Anxiety (OR 0.98; 95% CI 0.95, 1.02; *n* = 749), depression (OR 1.00; 95% CI 0.96, 1.05; *n* = 749) and satisfaction with life (OR 1.02; 95% CI 0.996, 1.04; *n* = 745) at baseline were not associated with weekly frequency of using the WW mobile phone app (Table 5). Every one unit of baseline global quality of life was associated with 69% lower odds of reporting high levels of weekly engagement with the WW mobile phone app (OR 0.31; 95% CI 0.15, 0.64; *n* = 730).

### Association of mental health with study visit attendance

#### Association of mental health with attendance at 3-month study visit

Every one unit of baseline global quality of life (OR 1.95; 95% CI 1.20, 3.17; *n* = 1209) and baseline satisfaction with life (OR 1.02; 95% CI 1.00, 1.04; *n* = 1209) were associated with a 95% and 2% higher odds of attending the 3-month study visit respectively. Conversely, every one unit of baseline anxiety (OR 0.95; 95% CI 0.92, 0.98; *n* = 1209) and baseline depression (OR 0.93; 95% CI 0.89, 0.97; *n* = 1209) were associated with a 5% and 7% lower odds of attending the 3-month study visit (Table 6).

**Table 6:** Influence of baseline mental health on attendance at study follow-up visits: Results are presented from unadjusted and mutually adjusted logistic regression models. Mutually adjusted models are adjusted for baseline mental health variables that were statistically significant in unadjusted models. Baseline mental health variables that were not statistically significant in unadjusted models were not adjusted for in mutually adjusted models. All models were controlled for randomised group and clustering by general practice

**Mental health at baseline:**	**Attendance at 3-month study visit (Odds ratio (95% CI))**	
	**Unadjusted logistic regression (*n*** **= 1209)**	**Mutually adjusted logistic regression (*n*** **= 1196)**
**Global quality of life**	1.95 (1.20, 3.17)	1.25 (0.67, 2.30)
**Satisfaction with life**	1.02 (1.00, 1.04)	0.99 (0.97, 1.02)
**Anxiety**	0.95 (0.92, 0.98)	0.99 (0.94, 1.04)
**Depression**	0.93 (0.89, 0.97)	0.94 (0.87, 1.01)
**Mental health at baseline:**	**Attendance at 12-month study visit (Odds ratio (95% CI))**	
	**Unadjusted logistic regression (*n*** **= 1227)**	**Mutually adjusted logistic regression (*n*** **= 1224)**
**Global quality of life**	1.48 (0.93, 2.37)	*^^*
**Satisfaction with life**	1.02 (1.00, 1.04)	1.01 (0.98, 1.03)
**Anxiety**	0.94 (0.91, 0.97)	0.96 (0.92, 1.00)
**Depression**	0.94 (0.91, 0.98)	0.97 (0.93, 1.02)
**Mental health at baseline:**	**Attendance at 24-month study visit (Odds ratio (95% CI))**	
	**Unadjusted logistic regression (*n*** **= 1237)**	**Mutually adjusted logistic regression (*n*** **= 1224)**
**Global quality of life**	1.61 (0.97, 2.66)	*^^*
**Satisfaction with life**	1.03 (1.00, 1.05)	1.01 (0.99, 1.04)
**Anxiety**	0.94 (0.91, 0.97)	0.95 (0.91, 0.98)
**Depression**	0.95 (0.91, 1.00)	1.00 (0.95, 1.06)
**Mental health at baseline:**	**Attendance at 60-month study visit (Odds ratio (95% CI))**	
	**Unadjusted logistic regression (*n*** **= 1237)**	**Mutually adjusted logistic regression (*n*** **= 1196)**
**Global quality of life**	2.02 (1.27, 3.23)	1.41 (0.83, 2.38)
**Satisfaction with life**	1.03 (1.00, 1.05)	1.01 (0.98, 1.04)
**Anxiety**	0.95 (0.92, 0.98)	0.98 (0.95, 1.02)
**Depression**	0.94 (0.91, 0.97)	0.97 (0.92, 1.03)

*Note:* ^^ Not included in mutually adjusted model

*Abbreviations*: CI Confidence interval

Baseline quality of life, satisfaction with life, anxiety, and depression were not associated with attendance at the 3-month study visit in the mutually adjusted model (Table 6).

#### Association of mental health with attendance at 12-month study visit

Baseline global quality of life was not associated with attendance at the 12-months study visit (OR 1.48; 95% CI 0.93, 2.37; *n* = 1227). A unit higher baseline satisfaction with life score was associated with 2% higher odds of attending the 12-month study visit (OR 1.02; 95% CI 1.00, 1.04; *n* = 1227). A unit higher anxiety (OR 0.94; 95% CI 0.91, 0.97; *n* = 1227) and baseline depression (OR 0.94; 95% CI 0.91, 0.98; *n* = 1227) score at baseline were associated with a 6% lower odds of attending the 12-month study visit (Table 6). Baseline satisfaction with life, anxiety, and depression were not associated with attendance at the 12-month study visit in the mutually adjusted model (Table 6).

#### Association of mental health with attendance at 24-month study visit

Baseline global quality of life was not associated with attendance at the 24-months study visit (OR 1.61; 95% CI 0.97, 2.66; *n* = 1237). A unit higher baseline satisfaction with life score was associated with a 3% increase in odds of attending the 24-month study visit (OR 1.03; 95% CI 1.00, 1.05; *n* = 1237). A unit higher baseline anxiety (OR 0.94; 95% CI 0.91, 0.97; *n* = 1237) and depression (OR 0.95; 95% CI 0.91, 0.997; *n* = 1237) scores were associated with a 6% and 5% lower odds of attending the 24-month study visit (Table 6).

Baseline satisfaction with life and baseline depression did not remain associated with attendance at the 24-month study visit when adjusted for baseline anxiety and depression (Table 6). When adjusted for baseline satisfaction with life and depression, a unit in baseline anxiety was associated with a 5% decrease in odds of attending the 24-month visit (OR 0.95; 95% CI 0.91, 0.98); *n* = 1196).

#### Association of mental health with attendance at 60-month study visit

A unit higher baseline global quality of life (OR 2.02; 95% CI 1.27, 3.23; *n* = 1237) and satisfaction with life (OR 1.03; 95% CI 1.00, 1.05; *n* = 1237) scores were associated with a 102% and 3% increase in odds of attending the 60-month study visit. Conversely, a unit higher baseline anxiety (OR 0.95; 95% CI 0.92, 0.98; *n* = 1237) and depression (OR 0.94; 95% CI 0.91, 0.97; *n* = 1237) scores were associated with a 5% and 6% decrease in odds of attending the 60-month study visit (Table 6). Baseline quality of life, satisfaction with life, anxiety, and depression were not associated with attendance at the 60-month study visit in the mutually adjusted model (Table 6).

## Discussion

In this study, we investigated whether participants’ mental health was associated with the rate of attendance and engagement with the behavioural weight management programme (WW) and trial (WRAP). Lower levels of depression symptoms and higher scores for satisfaction with life at baseline were associated with higher rates of attendance at WW sessions (i.e., higher rates of programme attendance). Similarly, adults with obesity who reported experiencing fewer symptoms of depression and anxiety and a higher satisfaction with life at baseline were more likely to report higher engagement with the programme resources (WW e-tools and mobile phone app). We also found that those reporting greater quality of life at baseline were likely to have lower engagement with the WW mobile phone app than those with poorer quality of life, however this association was not consistent across study findings. Higher scores for quality of life and satisfaction with life and fewer symptoms of depression and anxiety were also associated with higher attendance at all study visits up to 5 years follow-up.

Overall, we found that adults with obesity who self-reported being more mentally healthy at baseline were more likely to attend programme sessions, engage with programme resources, and attend study follow-up assessments. Previous research has reported that low levels of attendance and engagement in behavioural weight management trials are associated with decreased likelihood of achieving clinically significant weight loss, consequently reducing the likelihood of gaining the associated health benefits [9–11]. These findings highlight an emerging research priority to explore how to maximise the engagement and retention of people experiencing poorer mental health.

It is important to note, however, that study findings should be interpreted with consideration to the loss of significance in mutually adjusted models and the magnitude of the effect sizes presented. Many significant associations in univariable models became non-significant when mutually adjusted, potentially due to the mental health-related exposure variables being moderately correlated (Additional file 1: Table S1). Cross-correlation of exposure variables found correlations to range between small and large, with measures of anxiety and depression most highly correlated. Future research may consider the use of a composite indicator by combining individual measures into a single index, therefore reducing the impact of multicollinearity. The effect sizes of the associations between mental health and attendance and engagement were considered to be small, suggesting a small increase in risk of reporting lower attendance and engagement when reporting poor mental health at baseline [48]. However, these changes may still have a meaningful effect on the effectiveness of the behavioural weight management programme and trial. More research is needed to confirm the size of effect and investigate the potential impact on programme effectiveness and health outcomes.

Previous systematic review evidence has found no consistent psychological factors to be associated with attendance and engagement, but recommended further research into this area [7]. In the current study, we found poorer rates of attendance and engagement in those with poor mental health scores at baseline, aligning with the findings of McLean and colleagues who reported lower attendance among participants with higher levels of anxiety or depression [33]. Furthermore, current findings also align with previous research reporting those with higher levels of anxiety or depression are more likely to report worse attendance and engagement with the weight management trial [13, 34]. Some previous research, however, has reported finding mental health to not be associated with attendance and engagement [14, 35], contrasting with the findings of the current study. This may be due to the differing classification of mental health within different studies. For example, Funderburk and colleagues defined mental health as diagnosed versus non-diagnosed with mental illness [35], whereas we considered mental health as a symptom-continuum which appreciates that participants can experience one or more symptoms of mental illness without meeting diagnostic criteria [30, 31]. This approach was selected as it is associated with reduced stigma, and enables the investigation of a broader range of mental health outcomes [30–32].

There are many reasons why mental health might influence attendance and engagement with a behavioural weight management programme. Having poor mental health can make participation and engagement difficult and, in particular, can make it difficult to engage in things that may interfere with self-soothing coping strategies (e.g., emotional eating) [49]. Many adults with obesity seek social support and socialisation from WW groups [50]. For those living with poor mental health, forming connections with others can be impeded by an increased likelihood to withdraw/isolate, be less vulnerable with others when forming connections, and be more likely perceive others to judge them poorly (i.e., self-conscious/lacking self-esteem) [50]. In turn, those living with obesity and mental ill-health are likely to experience greater difficulties to connect with peers at the WW group than those who are mentally healthy, thus reducing the likelihood of programme attendance as their hopes and expectations of social support are not met. Previous negative experiences with healthcare providers (e.g., experiencing blame, shame, and discrimination for weight and/or mental health) may have reduced trust in services and healthcare staff, resulting in reduced engagement with services [49, 51]. Participants may also find that lack of immediate weight changes emphasises feelings of failure and shame, reduces their confidence in services’ effectiveness [52–55], and results in feelings of disappointment with the programme content [53]. Understandably, these feelings may decrease their motivation to attend the programme sessions.

Future research could use qualitative approaches to explore the mechanisms underpinning why those with poor mental health are less likely to attend and engage with behavioural weight management programmes. The mechanisms influencing programme attendance and engagement may be individual-level, societal-level, or operate across multiple levels. Understanding of these mechanisms would enable development of approaches to combat this issue. Furthermore, future research might explore whether tailoring programmes for those with obesity and poor mental health may result in improved programme attendance and engagement. For example, the RAINBOW trial provided a tailored weight management programme for persons with obesity and depression, finding modest improvements in both weight and depression symptoms [56]. We are unaware, however, if the tailored programme resulted in greater attendance and engagement rates than similar individuals attending a standard behavioural weight management programme.

The findings of the current study suggest that follow-up participant samples are less likely to include people with poorer mental health at baseline. Potential explanations may be higher levels of anxiety regarding clinical assessments (e.g., blood-sampling), lack of confidence in trial effectiveness (e.g., lack of immediate results), discomfort answering personal questionnaires (e.g., psychological assessments), and perceived participant burden (e.g., unhappy with length of questionnaires about mental health) [57–59]. Previous evidence from behavioural weight management trials should not be discarded, however caution should be taken when interpreting findings. In future trials, researchers should consider implementing a participant retention strategy that supports those with poorer mental health at baseline.

### Strengths and limitations

It is uncommon for behavioural weight management trials to measure and report mental health outcomes [6]. This secondary analysis of the WRAP trial benefited from the inclusion of multiple mental health outcomes at each assessment. Study findings were limited to those mental health measures implemented in the WRAP trial, the primary focus of which was the impact on weight and related outcomes [36]. Mental health measures not collected in this trial may further explain participant attendance and engagement rates, such as social support, stress, loneliness, self-esteem, and self-efficacy. Future research should aim to measure a wide range of mental health outcomes, but this must be balanced against participant burden.

A limitation was the large proportion of missing data, specifically for attendance at WW sessions and engagement with the WW resources. We had pre-specified a sensitivity analysis using multiple imputation, but also pre-specified that this would not be appropriate if the level of missingness were 25% or more (as it was for the programme attendance and engagement analyses), and would not be necessary if the level of missingness were less than 5% (as it was for the analysis of study attendance).

In addition, it is worth noting that engagement with WW digital resources and the mobile phone app was assessed using a self-report questionnaire and is therefore subject to potential error and bias, such as recall error and social desirability bias. To increase the accuracy of engagement data going forward, we recommend the use of objective measures either in replacement or in combination with self-report measures.

Despite these limitations, this study contributes new evidence to the field by seeking to understand the factors impacting attendance and engagement in behavioural weight management trials. To our knowledge, this study is one of the first to explicitly focus on the impact of mental health on programme and study attendance and engagement. We investigated the impact of participant mental health on numerous attendance and engagement factors and found relatively consistent evidence across factors. The aims of this secondary analysis were determined a priori, with a comprehensive statistical analyses plan determined prior to obtaining the trial data. The WRAP trial benefits from the randomised controlled trial design and minimal eligibility criteria that increase the representative nature of the baseline participant sample relative to the general population of adults with obesity in the UK.

## Conclusions

This study shows that adults with obesity attending a behavioural weight management programme are less likely to attend programme sessions, engage with programme resources, and attend study follow-up visits if they report higher levels of depression and anxiety, and lower scores for quality of life and satisfaction with life at baseline. The small effect sizes reported suggest a small increase in odds of low attendance and engagement in those experiencing poor mental health at baseline, however the changes may still have a meaningful effect on programme effectiveness and trial completion. Lower attendance and engagement are associated with reduced likelihood of achieving clinically significant weight loss and the associated health benefits, highlighting the importance of maximising participant attendance and engagement in behavioural weight management programmes. Future research should explore the effectiveness of targeted strategies to maximise attendance and engagement in those reporting poorer mental health upon entering a behavioural weight management programme or trial. Additionally, researchers should ensure to apply appropriate caution when interpreting trial findings, and sufficiently control for potential bias due to mental health when conducting trial analyses.

## Supplementary Information

**Additional file 1.**

## Data Availability

The dataset analysed during the current study is not publicly available. Participant consent allows for data to be shared in future analyses with appropriate ethical approval, and the host institution have an access policy (https://www.mrc-epid.cam.ac.uk/wp-content/uploads/2019/02/Data-Access-Sharing-Policy-v1-0_FINAL.pdf) so that interested parties can obtain the data for replication or other research purposes that are ethically approved. Data access is available from the senior author, who is also the principal investigator of the WRAP trial, upon reasonable request (ala34@cam.ac.uk).

## Abbreviations

A-Level: Advanced Level Qualification
BI: Brief intervention
BMI: Body mass index
CI: Confidence Interval
GCSE: General Certificate of Secondary Education
CP12: 12-weeks commercial weight management programme
CP52: 52-weeks commercial weight management programme
HADS: Hospital Anxiety and Depression Scale
IMD: Index of Multiple Deprivation
IRR: Incidence Rate Ratio
OR: Odds Ratio
SD: Standard Deviation
SLQ: Satisfaction with Life Questionnaire
WRAP: Weight loss Referrals for Adults in Primary care trial
WW: (formerly) Weight Watchers
