# Supplementary material for: The impact of participant mental health on attendance and engagement in a trial of behavioural weight management programmes: secondary analysis of the WRAP randomised controlled trial
Source: Int J Behav Nutr Phys Act. 2021 Nov 7;18:146. doi: 10.1186/s12966-021-01216-6 (PMC8574009; doi:10.1186/s12966-021-01216-6)
Supplement: Supplementary file 1 — Additional file 1. [file 12966_2021_1216_MOESM1_ESM.docx]

**The impact of participant mental health on attendance and engagement in a trial of behavioural weight management programmes: Secondary analysis of the WRAP trial.**

**ADDITIONAL FILE 1**

Rebecca A. Jones ([rj397@cam.ac.uk](mailto:rj397@cam.ac.uk)), Julia Mueller, Stephen J. Sharp, Ann Vincent, Robbie Duschinsky, Simon J. Griffin, Amy L. Ahern.

Table S1. Correlation matrix of mental health-related exposure variables.

|  | **Global quality of life** | **Satisfaction with life** | **Anxiety** |
| --- | --- | --- | --- |
| **Satisfaction with life** | 0.27 | / | / |
| **Anxiety** | -0.34 | -0.42 | / |
| **Depression** | -0.43 | -0.50 | 0.63 |
| *Note: Statistics presented are Pearson correlation coefficients.* | | | |
